# Cysteine-Functionalized Chitosan Magnetic Nano-Based Particles for the Recovery of Light and Heavy Rare Earth Metals: Uptake Kinetics and Sorption Isotherms

**DOI:** 10.3390/nano5010154

**Published:** 2015-02-04

**Authors:** Ahmed A. Galhoum, Mohammad G. Mafhouz, Sayed T. Abdel-Rehem, Nabawia A. Gomaa, Asem A. Atia, Thierry Vincent, Eric Guibal

**Affiliations:** 1 Ecole des mines d’Alès, Centre des Matériaux des Mines d’Alès, 6 avenue de Clavières, F-30319 Alès cedex, France; 2 Nuclear Materials Authority, P.O. Box 530, El-Maadi, Cairo, Egypt; E-Mails: mahfouznma@yahoo.com (M.G.M.); g_nabawia@hotmail.com (N.A.G.); thierry.vincent@mines-ales.fr (T.V.); 3 Chemistry Department, Faculty of Science, Ain Shams University, P.O. Box 11566 Ain Shams, Egypt; E-Mail: sayedth@sci.asu.edu.eg; 4 Chemistry Department, Faculty of Science, Menoufia University, P.O. Box 32511 Shebin El-Kom, Egypt; E-Mail: asemali2010@yahoo.com

**Keywords:** cysteine-grafting, rare earth metals, magnetic chitosan nanocomposites, sorption isotherms, uptake kinetics, thermodynamics

## Abstract

Cysteine-functionalized chitosan magnetic nano-based particles were synthesized for the sorption of light and heavy rare earth (RE) metal ions (La(III), Nd(III) and Yb(III)). The structural, surface, and magnetic properties of nano-sized sorbent were investigated by elemental analysis, FTIR, XRD, TEM and VSM (vibrating sample magnetometry). Experimental data show that the pseudo second-order rate equation fits the kinetic profiles well, while sorption isotherms are described by the Langmuir model. Thermodynamic constants (ΔG°, ΔH°) demonstrate the spontaneous and endothermic nature of sorption. Yb(III) (heavy RE) was selectively sorbed while light RE metal ions La(III) and Nd(III) were concentrated/enriched in the solution. Cationic species RE(III) in aqueous solution can be adsorbed by the combination of chelating and anion-exchange mechanisms. The sorbent can be efficiently regenerated using acidified thiourea.

## 1. Introduction

The recovery of heavy metals from dilute aqueous systems requires the development of new technologies for their concentration and separation [1]. Flocculation, coagulation, adsorption, ion exchange, membrane filtration, electrodeposition and chemical precipitation are the most conventional processes for the treatment of metal-bearing effluents. However, these techniques generally face economic or environmental constraints that make them ineffective for removing toxic or strategic metal ions at low or trace levels from aqueous waste streams. Adsorption is one of the most important physicochemical processes that has proved to be effective for metal recovery from dilute effluents [2].

Chitosan is a naturally abundant and biodegradable polysaccharide obtained by partial alkaline deacetylation of chitin: commercial chitosan is thus a copolymer of glucosamine and *N*-acetyl-d-glucosamine linked together by β (1→4) glycosidic bonds [3,4]. Most of its advantages are related to the wide availability of this renewable resource and its easy derivatization: it is readily chemically modified and can be physically conditioned under different forms [5]. In addition, this material is generally more hydrophilic than synthetic materials, such as polystyrene-divinylbenzene, polyethylene and polyurethane, which are commonly used as support for chelating and ion-exchange resins. Chitosan bearing amino groups can bind metal cations at near neutral pH by complexation/chelation on a free electronic doublet of nitrogen and metal anions by ion-exchange/electrostatic attraction on protonated amino groups in acid solutions [6]. Chitosan is a promising starting material for manufacturing new chelating/ion exchange resins [7]. The solubility of chitosan in acid media is a critical issue to be addressed for stable application; it is generally necessary to cross-link the biopolymer (by chemical modification) for extending the use of the biopolymer for a broader range of use (especially in terms of pH characteristics). It is often cross-linked to confer better microbiological and mechanical or chemical resistance [8]. On the other hand, the cross-linking of chitosan may contribute to reducing its ability to bind metal ions: in the case of Ln(III) sorption, it was attributed to the modification of chelating groups [9]. Therefore, novel chitosan resins bearing additional chelating moieties have been developed using the cross-linked chitosan resin as a starting support material [10]. A huge number of chitosan derivatives have been developed for the sorption of metal ions. The grafting of new functional groups on the backbone of chitosan increases the density of the sorption site and may change the sorption sites and the sorption mechanism, resulting in an increase of sorption capacity and a better selectivity for targeted metals [11].

Compared to conventional micron-sized supports used in separation process, nanometric sorbents possess quite good performance due to their high specific surface areas and the absence of internal diffusion resistance. However, the nano-adsorbents face serious drawbacks in terms of separation and recovery from treated solutions: they usually require sophisticated separation processes, such as very fine filtration or centrifugation. Magnetic nano-adsorbents offer interesting alternative for phase separation using an external magnetic field for the recovery of spent sorbents [12]. An additional advantage of these magnetic materials concerns their use in hazardous conditions, such as encountered in irradiated zones. Magnetic particles are usually composed of a magnetic core to ensure a strong magnetic response and a polymeric shell to provide favorable functional groups for sorption applications [13,14,15,16,17]. Alternatively, the magnetic core can be directly decorated with reactive groups through spacer arms [18]. In addition, iron (or other metal) oxide (or hydroxide) particles can bind metal ions [19,20], including rare earth elements (REEs) [21].

The REEs are the group of 17 chemical elements, including (a) two transition metal elements, scandium and yttrium; and (b) the 15 lanthanides (with atomic numbers 57–71). Rare earth elements are important in photo-electronic and metallurgical industries, as well as in nuclear energy programs. The demand for rare earths and their alloys as structural materials, fluxes and radiation detectors, diluents of plutonium, *etc.*, in nuclear technology is steadily increasing [22]. The designation “rare earths” refers to the elements of the periodic table known as “lanthanides”, further divided as a function of their atomic number into the “cerium group “(or “light” RE elements: La, Ce, Pr, Nd, Pm, Sm, Eu, Gd), and the “yttrium group” (or “heavy” RE elements: Y, Tb, Dy, Ho, Er, Tm, Yb, Lu) [23,24]. The challenge and the specificity of these elements are associated with the fact that (a) they do not naturally occur in their metallic form; and (b) they are difficult to separate from each other due to their very similar physicochemical properties (very close electronic configurations). Their trivalent state is the more stable form of RE ions in aqueous solution. Each lanthanide element has in its electronic configuration an inner shell with electrons in the 4f^n^ orbital shielded by an outer shell composed of electrons in orbitals 5s^2^, 5p^6^, 5d^1–10^, and 6s^2^. The differences among lanthanides are caused by the electrostatic effect associated with the increase of the shielded nuclear charge through the electron partial supply of the 4f orbital (which results in lanthanide contraction of the atomic and ionic radius along lanthanide series) [25]. This contraction is responsible for the low differences in their chemical properties, which allow lanthanide separation by fractionating methods. Traditionally, rare earth elements with similar chemical behaviors are first separated into a group before being further separated and purified. RE trivalent ions (Pearson hard acids: high oxidation state ions, species with low electronegativity and small size) tend to readily react with oxygen, nitrogen, sulfur, and phosphorus atoms (Pearson hard bases: electron donors, with high electronegativity and low polarizability). According to the theory of hard and soft acids and bases (HSAB) defined by Pearson, metal ions (depending on their hardness) will have a preference for complexing with ligands that have more or less electronegative donor atoms [26]. It is important to establish the affinity differences among selected elements to propose a process for lanthanide separation and purification through the sorption process [27]. One of the promising methods is the use of chelating or coordinating resins with covalently bound functional groups containing one or more donor atoms that are capable of directly forming complexes with metal ions. These polymers can also be used for a specific separation of one or more metal ions from solutions with different chemical environments [28]. In chelating resins, the functional group atoms that are most frequently used are nitrogen (e.g., N present in amines, azo groups, amides, nitriles), oxygen (e.g., O present in carboxylic, hydroxyl, phenolic, ether, carbonyl, phosphoryl groups) and sulfur (e.g., S present in thiols, thiocarbamates, thioethers). Usually, the anchored molecules contain nitrogen, oxygen or sulfur atoms, or a combination of them, acting as the basic centers that complex cations and allow selective extraction [29]. Several chelating ligands such as catechol, iminodiacetic acid, iminodimethyl-phosphonic acid, phenylarsonic acid, or serine [9], Ethylenediaminetetraacetic acid (EDTA) and/or diethylene triamine pentaacetic acid (DTPA) [10,30] and amino acids moieties (glycine, valine, leucine, and serine) [5] were used to functionalize crosslinked chitosan for the sorption of lanthanide metal ions.

In the present work, nano-magnetic particles were prepared using chitosan as the encapsulating material for embedding Fe_3_O_4_ nanoparticles (as the core material): the magnetic particles were *in situ* synthesized in the biopolymer. The composite material was chemically modified and functionalized through successive treatments with epichlorohydrin and cysteine (bringing the reactive functional groups). The structural, surface, and magnetic characteristics were investigated by elemental analysis, FTIR spectrometry, XRD and TEM analysis. The magnetic properties were measured using a vibrating-sample magnetometer (VSM). The sorption properties were investigated in batch tests on three different lanthanide ions: (a) “light” La(III) and Nd(III); and (b) “heavy” Yb(III). The sorption efficiency was evaluated through the influence of pH, sorption isotherms, and uptake kinetics. Thermodynamic parameters were also determined before investigating the regeneration of metal-loaded sorbent.

## 2. Results and Discussion

### 2.1. Synthesis of Sorbent Particles

A simple one-pot *in situ* co-precipitation method was used to synthesize magnetic chitosan nanoparticles. Chitosan precipitates in alkaline conditions simultaneously to the synthesis of magnetic iron particles (the reaction between Fe(II) and Fe(III) under alkaline conditions and under heating), resulting in the formation of composite chitosan-magnetic nano-based particles. The dropwise addition of NaOH leads to the formation of nanometric particles of a chitosan-magnetite composite [2].

**Figure 1:**
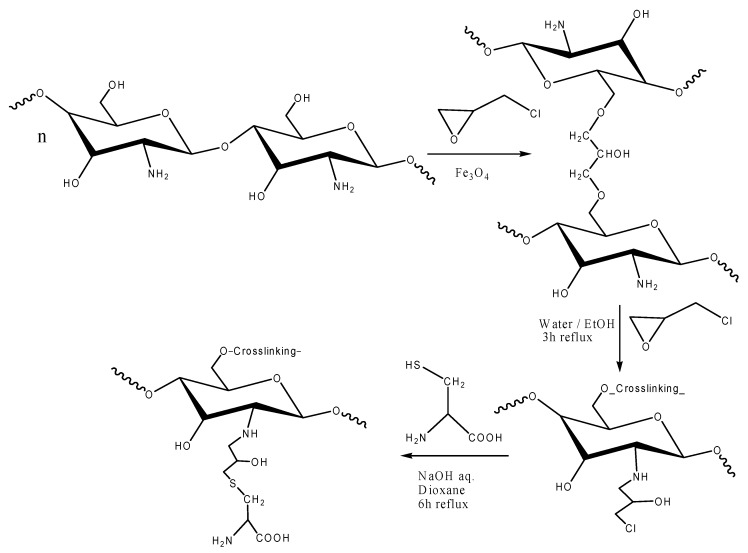
Scheme for the synthesis of cysteine-functionalized chitosan magnetic nano-based particles.

Chitosan-magnetite particles are chemically modified to prevent their dissolution in acidic media; however, aldehyde crosslinking may result in the loss of sorption capacity, because some amine groups are involved in the crosslinking reaction [8,31], so epichlorohydrin (or chloromethyloxirane) had been used as the crosslinking agent. Indeed, the crosslinking mono-functional agent is used to form covalent bonds with the carbon atoms linked to the hydroxyl groups of chitosan (associated with the rupturing of the epoxide ring and the release of a chlorine atom) [32]. Figure 1 shows the route for the synthesis of cysteine-functionalized chitosan magnetic nano-based particles.

### 2.2. Characterization of Sorbents

The C, H, N, and S contents in the cross-linked chitosan-magnetite material were 14.2%, 2.5%, 1.7% and 0%, respectively, while in the cysteine-grafted sorbent their values increased to 19.8%, 3.9%, 3.1% and 2.3%, respectively. The increases of carbon, hydrogen, nitrogen and sulfur contents clearly show the successful grafting of the cysteine moiety onto the cross-linked chitosan magnetite (through the epichlorohydrin spacer arm).

**Figure 2:**
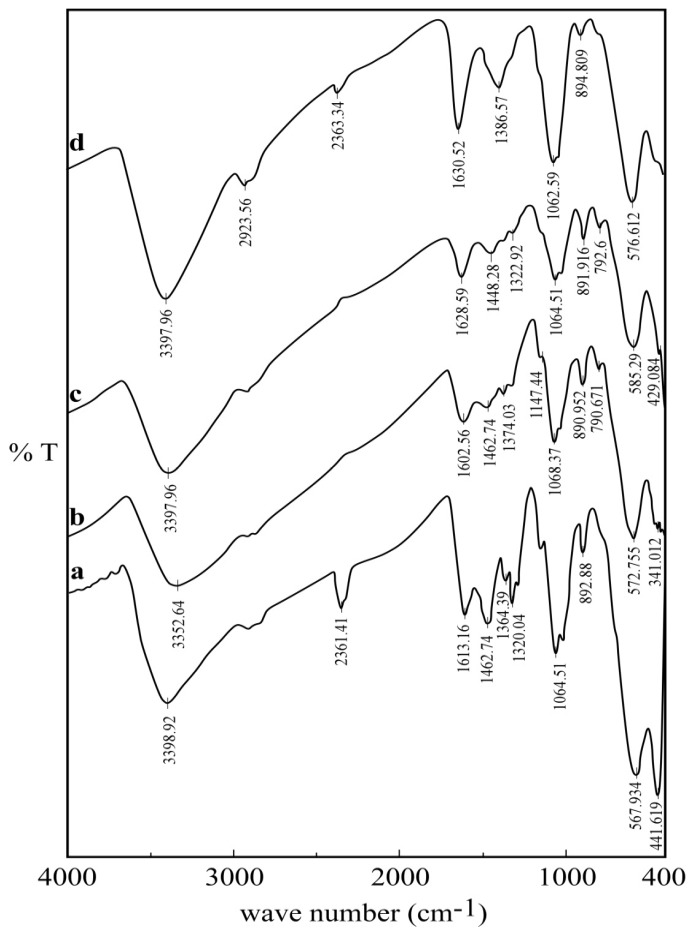
FTIR spectra of (**a**) chitosan-magnetite nanoparticles; (**b**) after cross-linking; (**c**) cross-linked chitosan magnetite with the spacer arm; and (**d**) cysteine-adsorbent.

To confirm the existence of the surface coating, the products obtained at each reaction step were characterized by FTIR spectra, as shown in Figure 2. The band at 568 cm^−1^ is assigned to the Fe–O stretching vibration of Fe_3_O_4_ [1,2]. A characteristic strong and broad band appeared at around 3399 cm^−1^, corresponding to the stretching vibration of the –OH group, the extension vibration of the N–H group and inter-hydrogen bonds of polysaccharides in the chitosan-magnetite spectra. The characteristic peaks of primary amine –NH_2_ appear at 3399 and 1613 cm^−1^. The bands at 1463 and 1364 cm^−1^ can be attributed to the C–O–C stretching and –OH bending vibrations, respectively. The absorption band at 893 cm^−1^ corresponds to the β-d-glucose unit [3]. The absorption bands around 1320 and 1065 cm^−1^ correspond to the stretching vibration of the primary –OH group and the secondary –OH group, respectively. However, the absorption intensities of –NH_2_ and –OH group (for the cross-linked material) are obviously lower than those on the chitosan-magnetite spectrum: the cross-linking reaction between chitosan and epichlorohydrin involves these two functional groups [3].

The introduction of spacer arms on the cross-linked chitosan is confirmed by the appearance of a new band at 792 cm^−1^ that can be attributed to –CH_2_–Cl stretching vibration (in comparison with the magnetic chitosan material) [33]. An additional band appears at 1631 cm^−^^1^; this band is characteristic of the (–COO^−^) carboxylate group vibration of the cysteine moiety [5]. In addition, the intensity of the band at 1387 cm^−1^ increases in the spectrum of the cysteine-type material; this is correlated with the introduction of additional amine groups. The values of amine group concentration of the cysteine-sorbent were found to be 3.53 mmol·g^−1^; this is about 24% more than in the spacer-arm-grafted cross-linked material.

**Figure 3:**
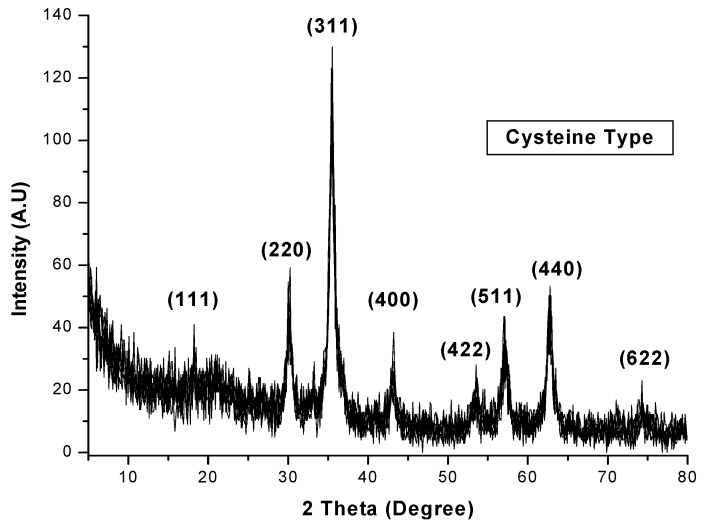
Powder X-ray diffraction (XRD) pattern of cysteine-sorbent nanoparticles.

The XRD pattern of cysteine-functionalized chitosan-magnetic nano-based particles is shown in Figure 3, together with the assignments of the different peaks representative of Fe_3_O_4_: (111), (220), (311), (400), (422), (511), (440) and (622). These peaks are consistent with the database in the JCPDS file (No. 65-3107) [1]. This confirms the existence of iron oxide particles (Fe_3_O_4_) with a spinel structure, which has magnetic properties and can be used for the magnetic separation and recovery of loaded particles. The half width at half maximum was used for calculating the size of nano-based particles through the Debye-Scherrer Equation [34]:

*D* = *k* λ/β cos θ (1)

where *D* is the average diameter of nanoparticles, λ is the wavelength of X-ray radiation (1.5418 Å), θ is the diffraction angle, *k* = 0.9 (shape parameter) and β is the full width at half maximum of the X-ray diffraction peaks. The crystal size has been found to be close to 13.5 nm (using index (311)).

**Figure 4:**
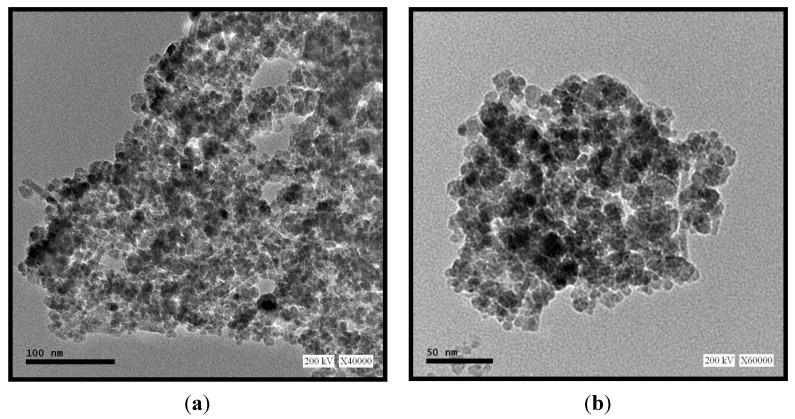
TEM micrographs (the scale bars are (**a**) 100 and (**b**) 50 nm, respectively).

The TEM image of the sorbent (Figure 4) shows that (a) the particles have a spherical morphology, and; (b) they are distributed in several classes of particles: 7–10 nm for the smallest and 20–25 nm for the largest. The sorbent particles are characterized by a partial aggregation that led to an average diameter of 150–250 nm. This aggregation may be attributed to the dipole-dipole magnetic attraction of nanoparticles. In addition, TEM also showed different contrasts on the photographs of chitosan-Fe_3_O_4_ composite particles: the dark areas can be attributed to the crystalline Fe_3_O_4_ core, while the bright or clear zones are associated with the chitosan coating. BET-analysis shows specific surface area close to 43 m^2^·g^−1^: this means 20–30 times the value usually reported for chitosan flakes: this is consistent with the values obtained for natural and synthetic magnetites [35]. This value is much smaller than the levels expected for nanoparticles: this means that even if some iron magnetic nanoparticles are not completely covered by the chitosan-based material this fraction of exposed iron particles is negligible compared to the coated particles. This is consistent with the TEM observation that shows the iron core coated by a thin layer of chitosan.

**Figure 5:**
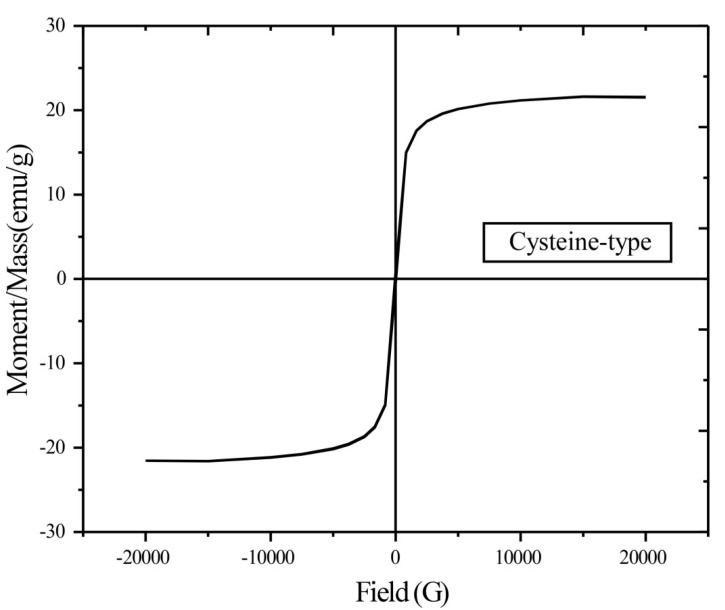
Magnetization curve of cysteine-functionalized chitosan magnetic nano-based particles.

Figure 5 shows the typical magnetization loop (hysteresis loop) for cysteine-functionalized chitosan magnetic nano-based particles. There is negligible remanence and coercivity; the chitosan-Fe_3_O_4_ nanoparticles can be described as super-paramagnetic nano-based particles.

This is consistent with the conclusions already reported by Kim *et al.* [36]: for nanometric materials below a critical size, the particles become individual single magnetic domains with remarkable superparamagnetic properties (*i.e.*, a large constant magnetic moment, a behavior similar to giant paramagnetic atoms with a fast response to the applied magnetic fields and negligible remanence and coercivity). The saturation magnetization was calculated to be about 21.51 emu·g^−1^ (21.51 A·m^2^·kg^−1^). This value is smaller than the levels reached by Chen and Wang [37] with a magnetic-chitosan composite (*i.e.*, around 35–40 emu·g^−1^), but a little higher than the values found in the literature (*i.e.*, 12–20 emu·g^−1^) for similar materials [12,13,38,39], and even more compared to systems prepared by coating of pre-formed magnetite particles with chitosan-based materials (*i.e.*, 6–13 emu·g^−1^) [17,40]. These values are significantly lower than the values obtained with pure Fe_3_O_4_ magnetic particles (*i.e.*, 50–70 emu·g^−1^); the decrease in the fraction of magnetic material, the diamagnetic contribution of the grafted copolymer layer and the crystalline disorder at the surface of the particles induced by the polymer layer may explain this reduction [13,17,40]. Weight loss on pristine chitosan and cysteine-based sorbent was determined at different temperatures (*i.e.*, 110 °C for water elimination, 400 °C, 600 °C and 800 °C) to follow the thermal degradation of organic material and evaluate the actual fraction of magnetite inorganic material (not shown): the fraction of magnetite was close to 49% in the final product; this is consistent with the expected values on the basis of the fraction of chitosan and magnetite introduced in the reactive media during sorbent synthesis. This may partially explain the substantial decrease of the saturation magnetization. In any case, the magnetic properties of synthesized hybrid materials make the sorbent easily recoverable with the help of an external magnetic field. This may be very helpful for solid/phase separation and/or handling the material in hazardous environments.

### 2.3. Sorption Properties

#### 2.3.1. Sorption as a Function of pH

Hereafter, the magnetic sorbent (*i.e.*, cysteine-type sorbent) has been carried out for the sorption of several metal cations (*i.e.*, La(III), Nd(III) and Yb(III)) from dilute sulfate solutions. It is well known that the sorption efficiency of sorbents can be affected by a variety of parameters: the pH is one of the most important parameters, especially for sorbents having acid-base properties (ion exchange or proton exchange characteristics).

**Figure 6:**
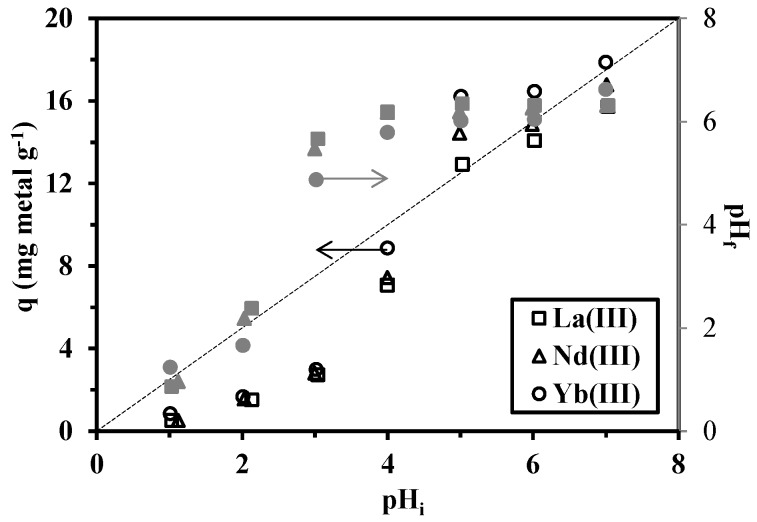
Effect of pH on the sorption of La(III), Nd(III) and Yb(III) ions using cysteine-functionalized chitosan magnetic nano-based particles (*C_i_* = 100 mg·L^−1^; *T* = 300 K; *t* = 4 h; *m* = 0.05 g; *V* = 100 mL).

The initial pH of the aqueous solution was varied between 1.0 and 7.0, controlled with either 0.5 M H_2_SO_4_ or 0.5 M NaOH. At a pH higher than 7.0, precipitation of Nd(III) and Yb(III) ions as M(OH)_3_ may spontaneously occur, making the interpretation of the sorption for metal concentrations higher than 100 mg·L^−1^ difficult. On the other hand, the inorganic magnetic material may also dissolve at a pH below 1.5. Figure 6 shows that sorption capacities increase with the increase in pH from 1.0 to 7.0, and there is a drastic increase at a pH of 5.0, while for higher pH values, the sorption capacity tends to stabilize. Figure 6 also shows pH variation during metal sorption. In the range pH 1–2, the final pH remained constant. When the initial pH was in the range 3–4, the equilibrium pH strongly increased up to 5–6. For initial values in the range of 6–7, the pH tended to stabilize around pH 6–6.5. Actually, the material has a kind of buffering effect around pH 5.5–6.5 when the initial value of the pH was set in the range of 3–7. This is probably due to the acid-base properties of chitosan (the pK_a_ of which depends on the degree of acetylation and varies between 6.3 and 6.8 for common chitosan samples [41]). For further experiments the pH was set to five to avoid any misinterpretation of the sorption performance that could be associated with metal hydrolysis or precipitation and to profit from both the optimum sorption performance and pH stabilization (pH variation of less than one pH unit).

In strong acidic solutions, both carboxyl groups and amino groups of the sorbent are protonated, resulting in a positively-charged surface for the sorbent. Therefore, the sorption capacities for La(III), Nd(III) and Yb(III) ions dramatically drop in low pH conditions; the sorption capacities are negligible at a pH close to 1.0 (Figure 6). At a low pH value, the coordinating atoms in the sorbent are partially protonated, as are the charged metal(III) species; this leads to repulsive electrostatic forces that limit the sorption of the metal on the cysteine-type material. However, this behavior is interesting since it allows predicting the possibility for the metal to be desorbed from the loaded-sorbent and the sorbent to be recycled using acid solutions (providing the acid-base stability of the sorbent is respected). As the pH increases, the protonated amine and carboxylic acid groups would gradually deprotonate. Therefore, the surface charge on the sorbent turns negative, which significantly enhances the electrostatic interaction between the sorbent and positively-charged metal ions [38].

Based on Figure 6, the sorption capacities (under selected experimental conditions) are found close to 16.2, 14.6 and 12.9 mg·g^−1^ for Yb(III), Nd(III) and La(III), respectively. In molar units, the sorption capacities’ range is close to 0.1 mmol·RE·g^−1^; this means that the three REs are equally sorbed by the material and that the differences in the ranking of the REs (between light REs, La(III) and Nd(III), and heavy RE, such as Yb(III)) are not sufficient for separating the metal ions.

The sorption of REs is clearly pH dependent and the cationic species can be sorbed through a chelating mechanism rather than an anion-exchange mechanism. This can probably be attributed to the presence of a free lone pair of electrons on nitrogen or sulfur that was suitable for coordination with metal ions to give the corresponding resin-metal complex. In addition, REE(III) ions can form chelates with the primary amino group (–NH_2_) and carboxyl group (–COOH), due to the limited steric hindrance. Moreover amino-based chelating resins may have ionic interaction properties through protonated amines (in acid solutions). On the other hand, sulfur is quite efficient for coordinating with metal ions [29], in a broad range of pH values.

#### 2.3.2. Uptake Kinetics

**Figure 7:**
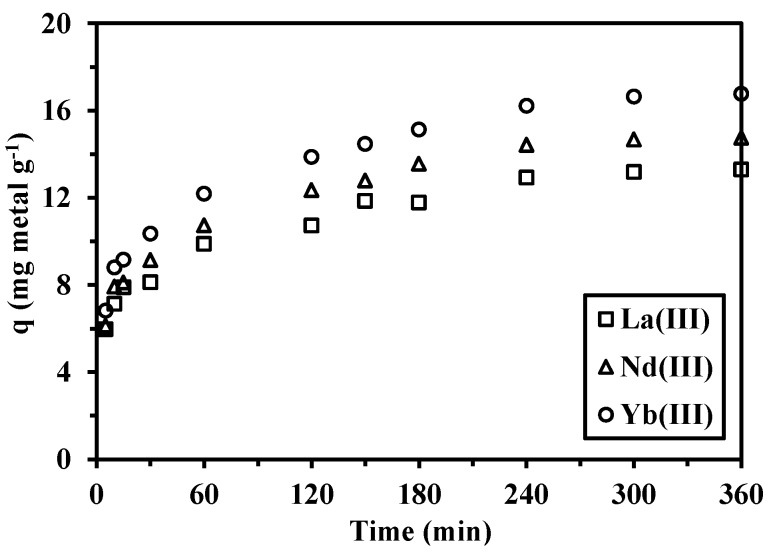
Effect of contact time on the adsorption of La(III), Nd(III) and Yb(III) ions (*C_i_* = 100 mg·L^−1^; *T* = 300 K; pH = 5; *m* = 0.05 g; *V* = 100 mL).

Sorption kinetics is another fundamental and significant issue for the evaluation of the potential of the sorbent for metal recovery. Figure 7 (displaying the plots of *q_t_* *versus* *t*) shows that, regardless of the metal, the sorption equilibrium is achieved within 4 h. The kinetic profiles have been analyzed by various models, such as the pseudo-first order rate equation (PFORE), the pseudo-second order rate equation (PSORE), the Elovich equation and the intraparticle diffusion kinetic models [42].

The linear forms of the PFORE and PSORE models are given in Equations (2) and (3), respectively:

(2) $$\log(q_{e}-q_{t})=\log q_{e}-\frac{k_{1}}{2.303}t$$

(3) $$\frac{t}{q_{t}}=\frac{1}{k_{2}q_{e}^{2}}+\frac{1}{q_{e}}t$$

where *q_e_* and *q_t_* (mg·g^−1^) are the sorption capacities at equilibrium and time *t* (min), respectively. *k*_1_ (min^−1^) and *k*_2_ (g·mg^−1^·min^−1^) are the rate constants of PFORE and PSORE, respectively.

The Elovich model is generally associated with chemisorption [43]. It was initially developed describing the kinetics of the chemical sorption of gases. However, recently, the Elovich equation was extensively used for modeling liquid phase sorption according to Equation (4):

q*_t_* = *A_E_* + *B_E_* ln *t* = 1/β ln(αβ) + 1/β ln *t* (4)

where *q_t_* is the amount of metal ion sorbed on the sorbents (mg·g^−1^) at time *t* (min), *A_E_* (mg·g^−1^) and *B_E_* (mg·g^−1^) are the Elovich constants, related to α (the initial sorption rate) and β (the function of surface coverage and activation energy).

The resistance to intraparticle diffusion is also an important step in the control of kinetics; especially for systems involving poorly porous materials, or large molecules. Several complex equations, such as the Crank equation, have been proposed for approaching diffusion models (derived from the Fick equation). In a first approximation, this equation was simplified with Equation (5) [43]:

*q_t_* = *k_int_*·*t*^0.5^ + *c* (5)

where *q_t_* (mg·g^−1^) is the amount of metal ions adsorbed at time *t* (min), and *k_int_* (mg·g^−1^·min^−0.5^) is the intraparticle diffusion constant.

The experimental data have been fitted by the aforementioned kinetic models: the parameters are all listed in Table 1 and Table 2. Based on the analysis of the correlation coefficients for the linear forms of the different kinetics models (Table 1), PSORE best fits the kinetics profiles for the sorption of La(III), Nd(III) and Yb(III) ions onto cysteine-functionalized chitosan magnetic nano-based particles. The fitting results of the pseudo-second order model are shown in Figure S1: solid lines fits the experimental data well. This means that the rate limiting step for sorption is probably the chemical adsorption rate that involves the valence forces through the sharing or exchange of electrons (*i.e.*, complexation, coordination and chelation). The metal binding within the first 30 min of contact is associated with physical adsorption, which is supposed to occur rapidly: this step represents about 62% of the total sorption. Thereafter, strong chemical interactions take place involving chemical bonding for charge neutralization, coordination and chelation [44]. Under selected experimental conditions (taking into account the metal concentration and sorbent dosage), the equilibrium is reached within 4 h: this contact time was selected for further equilibrium studies.

**Table 1:** Kinetics parameters of the pseudo-first order rate equation (PFORE) and the pseudo-second order rate equation (PSORE) for the sorption of La(III), Nd(III) and Yb(III) metal ions using cysteine-based magnetic-chitosan nanoparticles (*T*: 27 °C; pH: 5).

Metal ion	*q*_eq., exp._ (mg·g^−1^)	PFORE			PSORE		
		*k*_1_ × 10^2^ (min^−1^)	*q_e_* (mg/g)	*R*^2^	*k*_2_ × 10^3^ (g·mg^−1^·min^−1^)	*q_e_* (mg/g)	*R*^2^
La(III)	13.3	1.04	6.37	0.960	4.2	13.68	0.995
Nd(III)	14.8	1.15	7.75	0.984	3.7	15.27	0.996
Yb(III)	16.8	1.13	8.64	0.990	3.1	17.33	0.996

**Table 2:** Kinetics parameters of the Elovich and intraparticle diffusion models for the sorption of La(III), Nd(III) and Yb(III) metal ions using cysteine-based magnetic-chitosan nanoparticles (*T*: 27 °C; pH: 5).

Metal ion	Intraparticle diffusion			Elovich equation		
	*c*, mg·g^−1^	*k*_int._, mg·g^−1^·min^−0.5^	*R*^2^	*B_E_*	*A_T_*	*R*^2^
La(III)	4.26	0.56	0.832	1.75	2.90	0.979
Nd(III)	4.49	0.64	0.850	2.07	2.64	0.988
Yb(III)	5.02	0.86	0.855	2.36	2.86	0.991

Besides, the correlation coefficients for the pseudo-first order model and for the Elovich model were also higher than 0.92, but lower than the values obtained with PSORE, as shown in Table 1 and Table 2. This means that the pseudo-second-order model can be applied to predict the sorption kinetics and that the chemisorption is contributing to the kinetic control. Figure S2 shows that the relationship between *q_t_* and *t*^0.5^ is not linear (poor correlation coefficient): the intra-particle diffusion is not supposed to be the only rate-controlling step [45]. Actually, most sorption reactions take place through a multistep mechanism comprising [42]: (i) external film diffusion; (ii) intra-particle diffusion; and (iii) interaction between the sorbate and active site. In the present case, the resistance to film diffusion was significantly reduced by the appropriate agitation speed. The nanometric size of the sorbent limits the resistance to intraparticle diffusion: metal ions can readily diffuse to all reactive sites. Hence, the proper chemical reaction is supposed to play the major role in the control of the uptake kinetics.

#### 2.3.3. Sorption Isotherms

**Figure 8:**
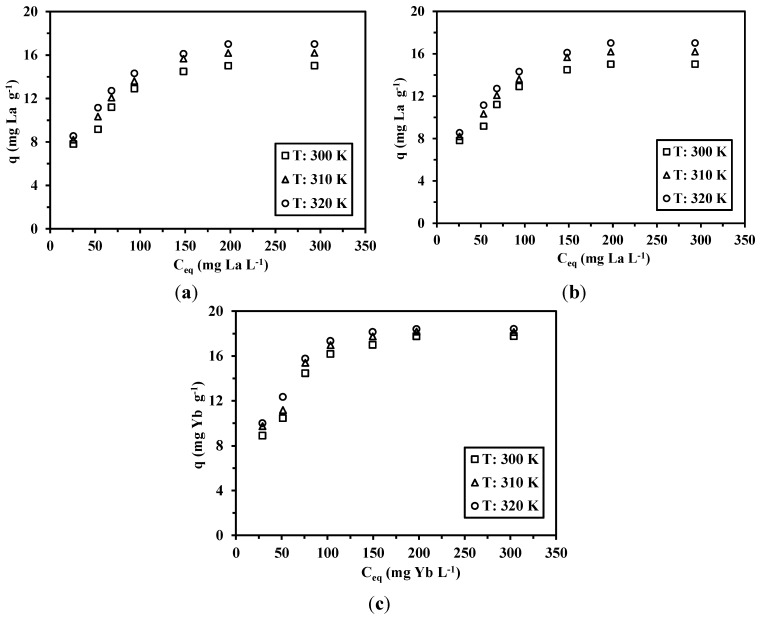
Adsorption isotherms for La(III), Nd(III) and Yb(III) ions at different temperatures. (*t* = 4 h; *T* = 300 K; pH = 5; *m* = 0.05 g; *V* = 20 mL).

Sorption isotherms are fundamental for understanding the interaction mechanisms and establishing both the maximum sorption capacities and the affinity of the sorbent for target solutes [46,47]. Different equations have been designed to model the distribution of the metal ions between liquid and solid phases (sorption isotherms), including the Langmuir, Freundlich, Temkin and Dubinin-Radushkevich (D-R) equations [46,47,48,49,50]. Though the fit of experimental data by a given equation does not necessarily means that the mechanisms associated with the model are verified, this may help in interpreting the sorption mechanism. Figure 8 shows the sorption isotherms for La(III), Nd(III) and Yb(III) using the cysteine-based sorbent at different temperatures, while Table 3 and Table 4 report the parameters of the different models.

All of the curves, regardless of the temperature and target metal, are characterized by the progressive increase of the sorption capacity followed by the saturation of the sorbent (plateau) that occurs for residual concentrations higher than 140–150 mg·metal·L^−1^. The asymptotic shape of the isotherm is consistent with the Langmuir equation (Equation (6)), contrary to the Freundlich equation (which supposes an exponential trend associated with the power function, Equation (7)):

(6) $$q=\frac{q_{m}bC_{eq}}{1+bC_{eq}}$$

where *q* is the amount of metal ions sorbed at equilibrium (mg·g^−1^), *C_eq_* is the equilibrium metal ion concentration in the aqueous solution (mg·L^−1^), *q_m_* is the maximum sorption capacity of the sorbent (mg·g^−1^), and *b* is the Langmuir sorption constant (L·mg^−1^), respectively.

(7) q=kF Ceq1n

where *k_F_* (L^1/*n*^·mg^1−1/*n*^·g^−1^) and n are the Freundlich constants.

**Table 3:** Parameters of the Langmuir and Freundlich equations for the sorption of La(III), Nd(III) and Yb(III) metal ions using cysteine-based magnetic-chitosan nanoparticles at different temperatures.

Metal ion	*T* (K)	*q*_m.,exp._ (mg·g^−1^)	Langmuir model			Freundlich model		
			*q*_m.,calc._	*K_L_* (L·mg^−1^)	*R*^2^	*n*	*K_F_*, (mg·g^−1^)	*R*^2^
La(III)	300	15.0	16.0	0.071	0.997	0.20	5.27	0.921
	310	16.2	17.1	0.079	0.998	0.20	5.84	0.953
	320	17.0	17.9	0.086	0.998	0.19	6.46	0.971
Nd(III)	300	15.3	16.0	0.106	0.999	0.19	5.92	0.934
	310	16.3	16.8	0.131	0.999	0.180	6.64	0.918
	320	17.1	17.6	0.144	0.999	0.17	7.17	0.920
Yb(III)	300	17.8	18.7	0.086	0.998	0.21	6.00	0.890
	310	18.1	18.9	0.120	0.998	0.18	7.40	0.871
	320	18.4	19.3	0.154	0.999	0.16	8.20	0.913

**Table 4:** Parameters of the Dubinin–Radushkevich (D–R) and Temkin equations for the sorption of La(III), Nd(III) and Yb(III) metal ions using cysteine-based magnetic-chitosan nanoparticles at different temperatures.

Metal ion	*T* (K)	*q_m_* (mg·g^−1^)	D–R Isotherm model			Temkin model		
			*K*_ad_ × 10^4^ (mol^2^·kJ^−2^)	*E_DR_* (KJ·mol^−1^)	*R*^2^	*A_T_* (L·mg^−1^)	*B_T_* (J·mol^−1^)	*R*^2^
La(III)	300	15.0	0.8	0.079	0.994	3.94	2.25	0.914
	310	16.2	0.6	0.091	0.939	5.00	2.35	0.945
	320	17.0	0.5	0.100	0.944	7.18	2.34	0.962
Nd(III)	300	15.3	1.0	0.071	0.968	6.67	2.19	0.948
	310	16.3	0.7	0.085	0.973	10.45	2.20	0.950
	320	17.1	0.5	0.100	0.981	13.62	2.23	0.955
Yb(III)	300	17.8	0.6	0.091	0.997	3.36	2.79	0.902
	310	18.1	0.4	0.112	0.997	10.41	2.44	0.876
	320	18.4	0.2	0.158	0.994	20.52	2.28	0.918

The Langmuir model supposes that: (a) all of the reactive sites are energetically equivalent (the same affinity for the target solute); (b) the sorption occurs as a monolayer; and (c) there are no interactions between the sorbed molecules. On the other hand, the empiric Freundlich model is generally associated with a heterogeneous distribution (and energy) of sorption sites with possible interactions between sorbed molecules, as well as a possible multilayer accumulation. The correlation coefficients (*R*^2^) of the linear form (obtained by plotting *C_eq_*/*q* *vs.* *C_eq_*, see Figure S3) for the Langmuir model were higher (and closer to one) than the values obtained for the Freundlich equation (Table 3). The values of Langmuir constants (*i.e.*, *q_m_* and *b*) are reported in Table 3. Both *q_m_* and *b* increased with increasing temperature: this means that metal binding is endothermic, regardless of the REE.

The dimensionless constant, *R_L_*, which reflects the essential characteristic of the Langmuir model, can be obtained from the constant *b*, according to Equation (8) [47]:

(8) $$R_{L}=\frac{1}{1+bC_{0}}$$

where *C*_0_ is the initial concentration of the metal ion. The calculated values of the dimensionless factor *R_L_* lie between 0.04 and 0.35 for La(III), 0.02 and 0.26 for Nd(III), and in the range of 0.02–0.29 for Yb(III), regardless of the concentration and the temperature. All of these *R_L_* values for the sorbent are smaller than 1.0: this is the first indication that the cysteine-based chitosan magnetic nano-based particles have a “favorable” sorption profile for La(III), Nd(III) and Yb(III).

The D–R isotherm model is usually employed for discriminating the nature of the sorption processes between physical and chemical mechanisms. The D–R isotherm equation is expressed by Equation (9a,b) [47,50]:

ln *q_eq_* = ln *q_DR_* − *K_DR_* ε^2^ (9a)

(9b) $$\mathrm{with}\;\varepsilon =RT\ln\left(1+\frac{1}{C_{eq}}\right)$$

where *q_DR_* is the theoretical saturation capacity, and ε is the Polanyi, *K_DR_* is related to the mean free adsorption energy per molecule of sorbate, *E_DR_* (kJ/mol). *E_DR_* provides information about the chemical or physical sorption, and can be determined according to Equation (9c):

*E_DR_* = (2*K_DR_*)^−1/2^ (9c)

Meanwhile, from the D–R isotherm, the plot of ln *q_eq_* *versus* ε^2^ gives a straight line with the slope *K_DR_* and the intercept ln *q_DR_*, as shown in Figure S4. The constants of the D–R isotherm (*q_DR_*, and *K_DR_*) are reported in Table 4.

The mean adsorption energy (*E_DR_*) corresponds to the transfer of the free energy of one mole of solute from infinity (in solution) to the surface of the sorbent. It is commonly accepted that physical sorption corresponds to mean adsorption energy below 8 kJ·mol^−1^, while chemical sorption requires mean adsorption energy greater than 8 kJ·mol^−1^ [47,50]. Regardless of the metal, the *E_DR_* for the cysteine-based magnetic-chitosan nanoparticles are in the range of 1–8 kJ·mol^−1^, the sorbent is supposed to bind La(III), Nd(III) and Yb(III) through a physisorption mechanism. In addition, the positive value of *E_DR_* confirms that the sorption process is endothermic, consistent with the improvement of the sorption capacities with temperature.

The Temkin model was also used to fit the experimental data. This model assumes that the free energy of sorption is a function of the surface coverage [47,48]. The isotherm is described by Equation (10).

(10) $$q=B_{T}\ln C_{eq}{+B}_{T}\ln A_{T}=\frac{RT}{\Delta Q}\ln C_{eq}+\frac{RT}{\Delta Q}\ln A_{T}$$

where *A_T_* is the Temkin equilibrium constant (L·mg^−1^), *B_T_* is a constant related to the surface heterogeneity of the adsorbent, Δ*Q* (−Δ*H*) is the variation of sorption energy (kJ·mol^−1^), T is the temperature (K) and *R* is the ideal gas constant (8.314 J·mol^−1^·K^−1^). Thus, the constants can be obtained from the slope and intercept of a straight-line plot of *q_eq_* *versus* ln *C_e_*. The constants of the Temkin model are listed in Table 4. The greater the constant *A_T_*, the higher the affinity of the sorbent for the solute; cysteine-based chitosan magnetic nano-based particles have a decreasing affinity according to Yb(III) > Nd(III) > La(III) based on *A_T_* values. It is noteworthy that *A_T_* increases as temperature does. The modeling of the uptake kinetics with the pseudo-second order rate equation (PSORE) suggested that chemisorption was the rate limiting step. On the other hand, the discussion of sorption isotherms led to the conclusion that metal binding occurs through physisorption (the value of the mean adsorption energies in the range 1–8 kJ·mol^−1^). This apparent contradiction in the interpretation of sorption mechanism can be explained by a dual mechanism that involves both ionic interaction (chemisorption) and electrostatic (physisorption) between the sorbent and RE metal ions. At a low metal concentration the solute is physically sorbed as a monolayer at the surface of the sorbent, while close to the saturation metal ions bind to the sorbent by coordination [47].

#### 2.3.4. Effect of Temperature—Thermodynamic Studies

The effect of temperature on the sorption of La(III), Nd(III) and Yb(III) by cysteine-functionalized chitosan magnetic nano-based particles was investigated at *T* = 300, 310 and 320 K, respectively. The values of *q_m_* are plotted *vs.* temperature in Figure S5. The amounts of metal ions sorbed gradually increased with increasing the temperature, as expected by the endothermic characteristics of the isotherms (values of *K_L_* and *E_DR_*, see Table 5). Wang *et al.* [45] attributed the improvement of affinity with temperature to the increase in the Lewis acid-base interaction between metal ions and the ligands.

**Table 5:** Thermodynamic parameters for the sorption of La(III), Nd(III) and Yb(III) metal ions using cysteine-based magnetic-chitosan nano-based particles at different temperatures.

Metal ion	*T* (K)	∆H° (kJ·mol^−1^)	∆S° (kJ·mol^−1^)	∆G° (kJ·mol^−1^)	*T*∆S° (kJ·mol^−1^)	*R*^2^
La(III)	300	7.85	0.103	−22.94	30.8	0.998
	310			−23.97	31.8	
	320			−24.99	32.8	
Nd(III)	300	12.04	0.120	−24.01	36.1	0.957
	310			−25.29	37.3	
	320			−26.49	38.5	
Yb(III)	300	23.43	0.158	−23.99	47.4	0.995
	310			−25.57	49.0	
	320			−27.15	50.6	

These experimental data (obtained at different temperatures) were used for calculating the thermodynamic parameters, such as standard Gibbs free energy change (ΔG°), enthalpy change (ΔH°) and entropy change (ΔS°). The thermodynamic parameters were then calculated from the van’t Hoff equation, and derived from Equations (11) and (12):

(11) $$\ln\;b=\frac{-\Delta H^{\circ }}{\mathrm{RT}}+\frac{\Delta S^{\circ }}{R}$$

**Figure 9:**
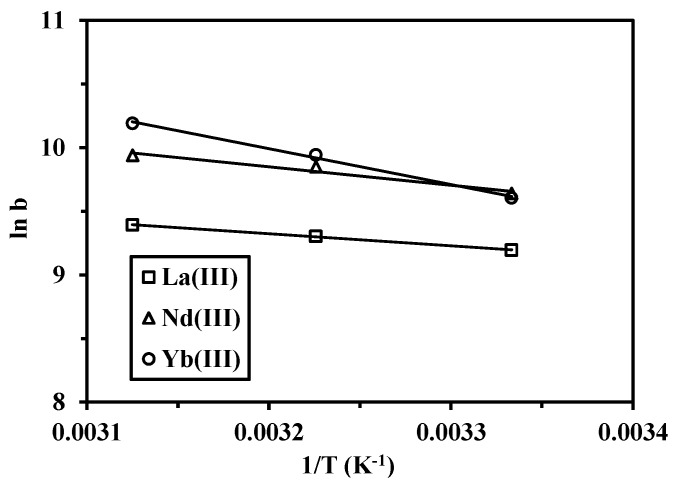
Van’t Hoff plots of ln *K_L_* against 1/*T*, for sorption of metal (III) ions.

ΔG° = ΔH° − TΔS°(12)

where *b* is the equilibrium constant, which can be obtained from Langmuir isotherm at different temperatures, and *T* is the absolute temperature (K). The values of enthalpy change (ΔH°) and entropy change (ΔS°) were obtained by plotting ln *b* *vs.* 1/*T* (Figure 9). The values of the thermodynamic constants (ΔH°, ΔS° and ΔG°) are reported on Table 5.

Table 5 confirms that the sorption reaction of La(III), Nd(III) and Yb(III) ions on cysteine-functionalized chitosan magnetic nano-based particles is endothermic (a positive value of ∆H°). The sorption enthalpy increases according to the sequence: La(III) < Nd(III) < Yb(III). The free energy is systematically negative: regardless of the metal, the value of free energy ranged between −23 and −27 kJ·mol^−1^. The decrease in the value of ∆G° with the increase in temperature shows that the reaction is enhanced at high temperature (confirmation of the endothermic nature of the sorption mechanism). The positive value of ∆S° may be related to the release of the water of hydration during the sorption process causing the increase in the randomness of the system. Metal ions in aqueous media are hydrated. When the ions get sorbed on the sorbent surface, water molecules previously bonded to metal ions are progressively released and dispersed in the solution; this results in an increase in the entropy of the system. A substantial difference is observed in entropy values between Nd(III) and Yb(III), on one side, and La(III), on the other side. Table 5 shows that |∆H°| is systematically lower than |T∆S°| in the studied temperature range. This means that the sorption process is dominated by entropic rather than enthalpy changes [51].

Table 6 reports the comparison of La(III), Nd(III) and Yb(III) sorption capacities for different sorbents. The direct comparison of sorption performance is difficult, since the experimental conditions may substantially differ; however, the data show that the cysteine-functionalized chitosan magnetic nano-based particles are lower than the levels reached with synthetic resins; they are comparable to the sorption capacities obtained with some biosorbents: the presence of a significant fraction of magnetite (about 50%) in the sorbent may explain the relative decrease of the sorption capacity. Indeed, the amount of reactive functional groups is halved.

**Table 6:** Comparison of the La(III), Nd(III) and Yb(III) sorption properties of different sorbents with cysteine-functionalized chitosan magnetic nano-based particles (CFCMNBP).

Sorbent	Metal	pH range	*q_m_* (mg·g^−1^)	Reference
Tangerine peel	La(III)	5	155	[52]
*Sargassum* sp.	La(III)	5	74–100	[53]
Magnetic alginate beads	La(III)	2.8	97	[16]
*Platanus orientalis* leaf	La(III)	4	29	[54]
Lewatit resins	La(III)	1.5–5	100–120	[55]
Chelating ion-exchange resin	La(III)	HCl/HNO_3_	188–240	[56]
Functionalized Amberlite XAD-4 resin	La(III)	6.1	49	[57]
CFCMNBP	La(III)	6	17	This work
EDTA:DTPA functionalized chitosan	Nd(III)	3–6	55	[10]
Phosphonic acid functionalized silica microspheres	Nd(III)	2.8	45	[58]
Ion imprinted polymer particles	Nd(III)	7.5	33	[59]
Phosphorus functionalized adsorbent	Nd(III)	6	160	[60]
Yeast cells	Nd(III)	1.5	10–12	[61]
Mordenite containing tuff	Nd(III)	5.5–6.5	13	[62]
CFCMNBP	Nd(III)	6	17	This work
*Sargassum*	Yb(III)	5	160	[27]
*Turbinaria conoides*	Yb(III)	4.9	34	[63]
*Pseudomonas aeruginosa*	Yb(III)	6–7	56	[64]
Imino-diacetic acid resin	Yb(III)	5.1	187	[65]
Gel-type weak acid resin	Yb(III)	5.5	266	[66]
CFCMNBP	Yb(III)	6	18	This work

#### 2.3.5. Metal Desorption and Resin Recycling

Generally, the sorbed metal ions can be desorbed using a selective eluent with the objective of concentrating/enriching the metal concentration. However, acids such as HCl and HNO_3_, may react with Fe_3_O_4_, which is the core component of magnetic chitosan nanoparticles. Ethylenediamine tetraacetic acid (EDTA) and thiourea are known as very strong chelating agents for many metal ions and are supposed to displace metal ions from reactive groups (based on the greater affinity of the metal for the ligands over reactive groups on sorbent particles).

Consequently, thiourea (0.5 M) acidified with a few drops of H_2_SO_4_ (0.2 M) was chosen as the eluant for metal ions, with a contact time of 1 h. The sorbent was tested for four successive sorption/desorption cycles: regardless of the metal, the sorption efficiencies and capacities slightly decreased along the cycles, but the variations remained quite low. In the worst case, the decrease in the sorption efficiency (compared to the first cycles) was less than 7% (Table 7). Though the mechanisms of desorption were not elucidated, it is supposed that metal is removed from the loaded sorbent through electrostatic and complexation mechanisms.

**Table 7:** Recycling of cysteine-based magnetic-chitosan nano-based particles for the sorption of La(III), Nd(III) and Yb(III) metal ions over four cycles.

Cycle	La(III)		Nd(III)		Yb(III)	
	*q_e_* (mg·g^−1^)	Ads. (%)	*q_e_* (mg·g^−1^)	Ads. (%)	*q_e_* (mg·g^−1^)	Ads. (%)
Cycle I	12.9	100.0	14.4	100.0	16.2	100.0
Cycle II	12.7	98.2	14.2	98.1	15.4	95.2
Cycle III	12.6	97.5	14.1	97.5	15.3	94.3
Cycle IV	12.6	97.1	14.0	96.9	15.2	93.8

#### 2.3.6. Sorption Selectivity

To investigate the selective sorption of Yb(III) ions from aqueous complex solutions, the sorption properties of cysteine-functionalized chitosan magnetic nano-based particles have been investigated using binary solutions containing equivalent concentrations of Nd(III) and Yb(III) (*i.e.*, nearly equimolar concentrations: *C*_0_ ≈ 0.23 mmol·Yb·L^−1^ (59.5 mg·Yb·L^−1^) and *C*_0_ ≈ 0.25 mmol·Nd·L^−1^) (53.8 mg·Nd·L^−1^). The results showed that the amount of Yb(III) sorbed (*i.e.*, 11.6 mg·Yb·g^−1^; *i.e.*, 0.067 mmol·Yb·g^−1^) is much higher than the amount of Nd(III) ions (*i.e.*, 5.0 mg Nd·g^−1^; *i.e.*, 0.035 mmol·Nd·g^−1^). At equilibrium, the fractions of Yb(III) and Nd(III) on the sorbent reached 69.7% and 30.3%, respectively. This means that the sorbent has a greater affinity for the heavy RE metal ion, Yb(III), compared to the light RE metal ion, Nd(III). The sorbent is enriched in Yb(III), while Nd(III) concentrates in the aqueous phase. This is positive, but not sufficient for achieving perfect metal separation.

Another important issue, which was not investigated in this study, concerns the effect of the presence of anions, such as bicarbonate or sulfate. It is well known that the presence of ligands (for example, lactate) may influence the selective binding of REEs (this may contribute to increase the differences in the chemical behavior of REEs) [67]. The key parameter is the speciation of metal ions in the presence of ligands, especially when ion-exchange mechanisms are involved: the formation of anionic complexes may increase the sorption properties of protonated reactive groups for anionic complexes. For example the presence of high concentrations of carbonate or phosphate leads to the formation of anionic complexes [68]. In some cases the presence of carbonate is required for producing ternary REE-carbonate-surface complexes to improve REE recovery [69]. On the other hand, the presence of nitrate, chloride or sulfate anions (in three-times excess of anion compared to REE) did not show a significant impact of REE sorption when using *Pseudomonas aeruginosa* biomass [64]. Schijf and Marshall [70] reported the decrease of sorption for yttrium and REEs on hydrous ferric oxide when increasing the ionic strength of the solution: they observe that at high ionic strength the sorption is less influenced by the pH and that the sorption decreases due to enhanced deprotonation of the sorbent surface. Tang and Johannesson [71] commented on the effect of carbonate/bicarbonate (in relation to pH) on the sorption of lanthanide onto sand: at a pH lower than 7.3, inorganic ligands did not significantly compete with surface sites for metal binding, while at a higher pH, lanthanide complexation with carbonate (to form anionic species) leads to simultaneous sorption of free and complexed REE forms. From the literature, it appears that both the pH and the presence of anions (at a high relative concentration) may influence the speciation of REEs and, consequently, their affinity for sorption on the reactive groups at the surface of composite nanoparticles. This requires a specific study for the application in complex effluents.

## 3. Experimental Section

### 3.1. Materials

Chitosan (90.5% deacetylation) was supplied by Sigma-Aldrich (France). Cysteine was obtained from Sigma-Aldrich, and epichlorohydrin (>98%), 1,4-dioxane (99.9%) and ethanol were purchased from Fluka Chemika AG (Germany). Sodium hydroxide solution (30%) was supplied by Chem-Lab. NV and all other chemicals were Prolabo products and were used as received.

### 3.2. Rare Earth Solutions and Analytical Procedures

La_2_O_3_, NdCl_3_ and YbCl_3_.6 H_2_O were purchased from Sigma-Aldrich and were burned off at 900 °C for 3 h. Stock solutions of rare earth ions La(III), Nd(III) and Yb(III) were prepared by mineralizing the corresponding salts in concentrated sulfuric acid under heating, before diluting with demineralized water until a final concentration of 1000 mg·L^−1^. The working solutions were prepared by appropriate dilution of the stock solutions immediately prior to use. The metal concentrations in both initial and withdrawn samples were determined by an Inductively Coupled Plasma Atomic Emission Spectrometer (ICP-AES JY Activa M, Jobin-Yvon, Longjumeau, France).

### 3.3. Sorbent Synthesis and Characterization

#### 3.3.1. Preparation of Cross-Linked Chitosan-Magnetite Nanocomposites

Chitosan-magnetite nanocomposites were prepared by chemical co-precipitation of Fe(II) and Fe(III) ions by NaOH in the presence of chitosan followed by treatment under hydrothermal conditions using a method derived from Namdeo and Bajpai [2]. Briefly, Chitosan (4 g) was dissolved in 200 mL (20%) acetic acid and mixed with FeSO_4_ and FeCl_3_ salts (added in a 1:2 molar ratio; *i.e.*, 6.62 g of FeSO_4_.7H_2_O and 8.68 g of FeCl_3_). The resulting solution was chemically precipitated at 40 °C by adding dropwise a 1 M NaOH solution under continuous stirring, at controlled pH (10–10.4). The suspension was heated at 90 °C for 1 h under stirring and finally recovered by decantation and magnetic separation. Then, a solution of 0.01 M epichlorohydrin containing 0.067 M sodium hydroxide was prepared (pH close to 10) and added to freshly prepared wet magnetite-chitosan nano-based particles in a ratio of 1:1. The mixture of chitosan-magnetite and epichlorohydrin was heated at 40–50 °C for 2 h under stirring [72]. Finally, the product (ii) was filtered and intensively washed with distilled water to remove any unreacted epichlorohydrin.

The amino acid moiety (cysteine) was introduced to the cross-linked chitosan magnetic material in two steps [5]. First, the cross-linked chitosan (ii) was suspended in a 150 mL ethanol/water mixture (1:1 *v*/*v*); then, epichlorohydrin (15 mL) was added to the suspension, and the mixture was refluxed for 4 h. After the reaction, the product (iii) was filtered and washed 3 times with ethanol and with ultrapure water to remove any residual reagent. Secondly, the washed product (iii) and cysteine (16 g) were suspended in dioxane (200 mL). The mixture was alkalinized to pH 9.5–10 using a 1 M NaOH solution; the mixture was refluxed for 6 h. After the reaction, the final product was filtered and washed 3 times with ethanol and with ultrapure water. Finally, the product was freeze-dried.

The amine content in the adsorbent was estimated using a volumetric method [73]: 30 mL of 0.05 M HCl solution were added to 0.1 g of the material and conditioned for 15 h on a shaker. The residual concentration of HCl was estimated through titration against 0.05 M NaOH solution using phenolphthalein as the indicator. The number of moles of HCl having interacted with amino groups and consequently the concentration of amino groups (mmol·g^−1^) was calculated from Equation (13):

Concentration of amino group = (*M*_1_ − *M*_2_) × 30/0.1 (13)

where *M*_1_ and *M*_2_ are the initial and final concentrations of HCl, respectively.

#### 3.3.2. Characterization Methods

Elemental composition of the resin (*i.e.*, C, H, S and N contents) was determined using a Heraeus CHN-O-Rapid elemental analyzer (Hanau, Germany). Powder X-ray diffraction (XRD) patterns were obtained at room temperature using an X-ray diffractometer Philips model PW 3710/31 (Eindhoven, The Netherlands), using the Cu K_α_ radiation in the range of 2θ = 10°–90°. The size and morphology of sorbent particles were obtained using a Hitachi H-800 transmission electron microscope (Hitachi, Japan). The magnetic property was measured on a vibrating-sample magnetometer (VSM) (Lake Shore 730T, Westerville, OH, USA) at room temperature. Functional groups of sorbent were analyzed by Fourier transform infrared spectrometry using a Nicolet Nexus 870 FTIR spectrometer (Thermo Electron Scientific Instruments Corporation, Madison, WI, USA) in the wavelength range 400–4000 cm^−1^ (using the KBr pellet technique). The BET specific surface area was determined through nitrogen adsorption isotherms using a Quantachrome NOVA 3200 (Boynton Beach, FL, USA) analyzer. The magnetite content was determined by measurement of the weight losses at different successive ignition temperatures (*i.e.*, 110, 400, 600 and 800 °C, exposure for 1 h).

#### 3.3.3. Sorption and Desorption Methods

Standard batch experiments were carried out by contact of 50 mg of sorbent (*m*) with 20 mL (*V*) aqueous metal ion solution (*C*_0_: 100 mg·metal·L^−1^; initial pH: 5) in a polypropylene centrifuge tube. The samples were agitated at 300 rpm (stirring speed) for 4 h (the temperature being set at 27 ± 1 °C). After magnetic separation, the residual metal ion concentration (*C_eq_*, mg·meta·L^−1^) in the aqueous phase was determined by ICP-AES, whilst the concentration of metal ions sorbed onto the sorbent (*q_eq_*, mg·metal·g^−1^) was obtained by the mass balance equation:

*q_eq_* = (*C*_0_ − *C_eq_*) × *V*/*m* (14)

Other experiments were based on the same procedures (varying the relevant parameter) for investigating the effect of pH, the influence of equilibration time (uptake kinetics), and the impact of metal concentration (sorption isotherms). Isotherms were obtained by contact of 50 mg of sorbent with 20 mL of a pH 5 solution containing the metal ions at different initial concentrations (25, 50, 75, 100, 150, 200 and 300 mg·L^−1^) under shaking (at a speed of 300 rpm) for 4 h. The experiments were performed at different temperatures (*i.e.*, 300, 310 and 320 ± 1 K).

The regeneration of the sorbent was tested by mixing the metal-loaded sorbent (sorbent dosage: 2.5 g·L^−1^; *C*_0_: 100 mg·metal·L^−1^, pH: 5; agitation speed: 300 rpm; contact time: 4 h) with the eluent (0.5 M thiourea solution slightly acidified with a few drops of 0.2 M sulfuric acid solution; pH close to 3) for 1 h under agitation. The sorbent was recovered by magnetic separation, and the residual concentration was analyzed by ICP-AES while the sorbent was recycled (after intensive washing). Four sorption/desorption cycles were successively carried out using standard procedures: the sorption capacity and sorption yield at each stage were compared (the desorption yield was not determined).

## 4. Conclusions

Cysteine-functionalized chitosan magnetic nano-based particles have been synthesized, characterized and used as a sorbent with a superparamagnetic property. The sorption properties have been efficiently tested for the sorption (and possible separation) of light (La(III) and Nd(III)) and heavy (Yb(III)) RE metal ions from aqueous solutions. The Langmuir isotherm model provided the best fit for the sorption isotherms of these three metal ions. The maximum sorption capacities at pH 5 were found to be 17.0, 17.1 and 18.4 mg·g^−1^ for La(III), Nd(III) and Yb(III) ions at 320 K, respectively. Thermodynamic parameters (ΔG° and ΔH°) indicate the spontaneous and endothermic nature of the sorption process, while the positive values of ∆S° indicate increased randomness due to metal sorption: the entropy of the system increases, probably due to the release of the water of hydration of metal ions after metal sorption. Finally, the sorbent can be regenerated with high efficiency by acidified thiourea as the eluent, and after three cycles, the adsorption capacities were not significantly reduced.

This sorbent showed promising properties; however, some parameters should be optimized. For example, the relative fraction of the magnetic core and chitosan layer should be varied in order to measure the impact of this parameter on the sorption and magnetization properties. Hence, increasing the modified chitosan content is expected to increase the sorption yield, but reduce the magnetization efficiency. The optimal formulation will be a compromise between the sorption and magnetization properties.

At this stage of the development of the process the feasibility of the synthesis of nano-based particles of chitosan has been demonstrated. The material can be readily modified by chemical grafting. The sorbent properties are not high enough, at this stage, for being competitive against more conventional systems. The magnetic nature of the sorbent particles is expected to make the handling and operating of the material in hazardous environments (such as radioactive environments) possible with minimized resistance to intraparticle diffusion (and enhanced kinetics; this means also the possibility of reducing the scaling up of the treatment unit by reducing required contact times).

## Supplementary Materials

Supplementary materials can be accessed at: http://www.mdpi.com/2079-4991/5/1/154/s1.
