# Supplementary material for: Cysteine-Functionalized Chitosan Magnetic Nano-Based Particles for the Recovery of Light and Heavy Rare Earth Metals: Uptake Kinetics and Sorption Isotherms
Source: Nanomaterials (Basel). 2015 Feb 4;5(1):154–79. doi: 10.3390/nano5010154 (PMC5312863; doi:10.3390/nano5010154)
Supplement: Supplementary file 1 [file nanomaterials-05-00154-s001.pdf]

Supplementary Information

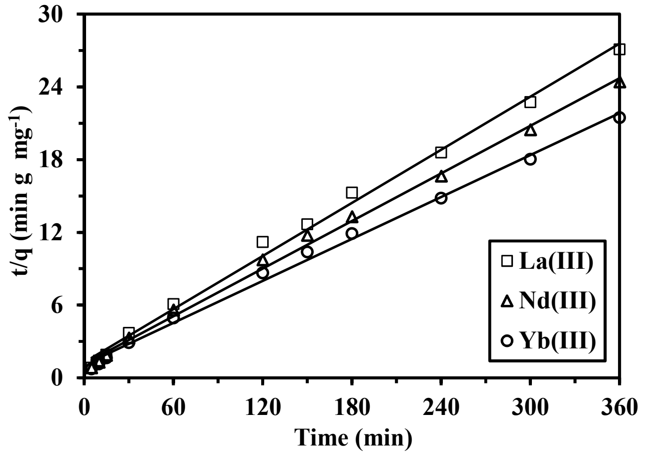

Figure S1. Pseudo-second order kinetic plots for the sorption of La(III), Nd(III) and Yb(III) ions. (C_i = 100 mg·L^{-1}; T = 300 K; pH = 5).

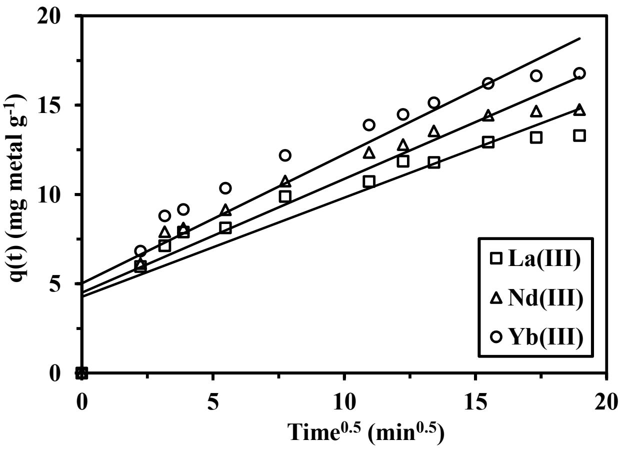

Figure S2. Intraparticle diffusion kinetic plots for the sorption of La(III), Nd(III) and Yb(III) ions. (C_i = 100 mg·L^{-1}; T = 300 K; pH = 5).

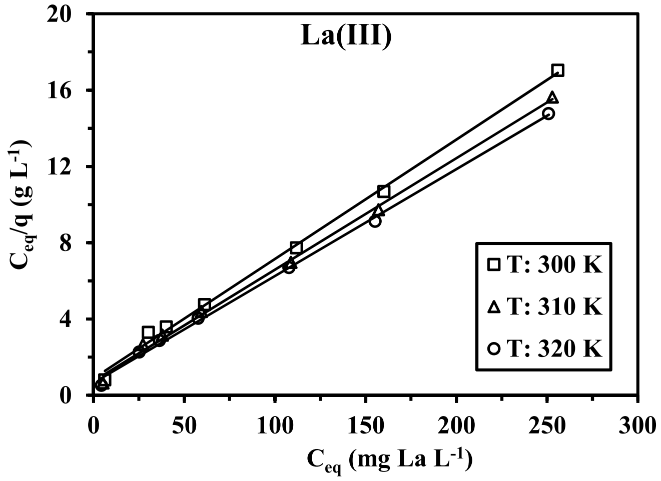

Figure S3. *Cont.*

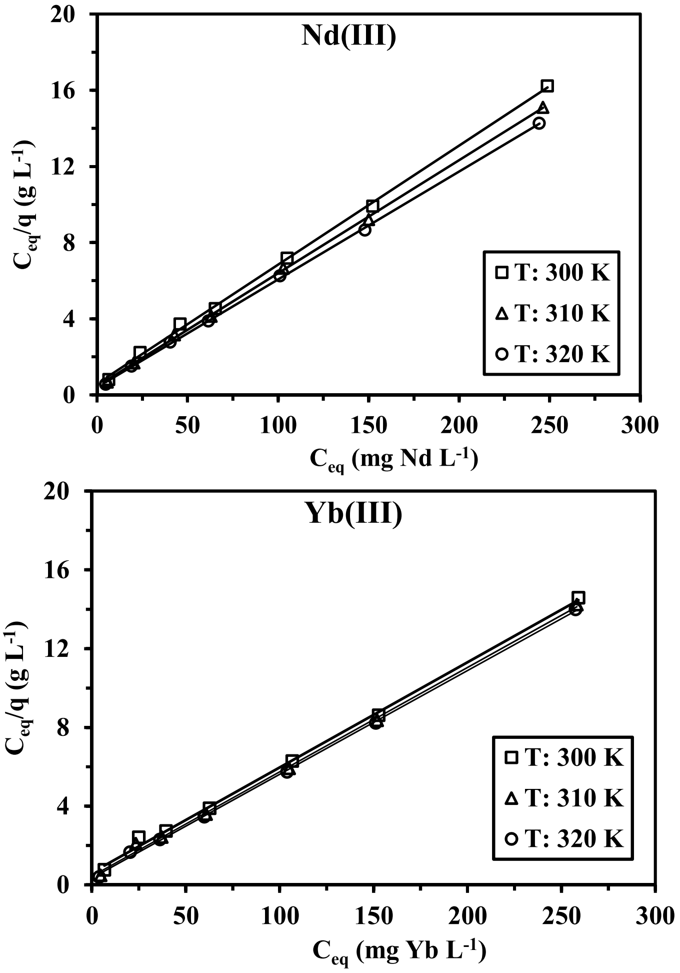

Figure S3. Langmuir plots for sorption of La(III), Nd(III) and Yb(III) ions at different temperatures.

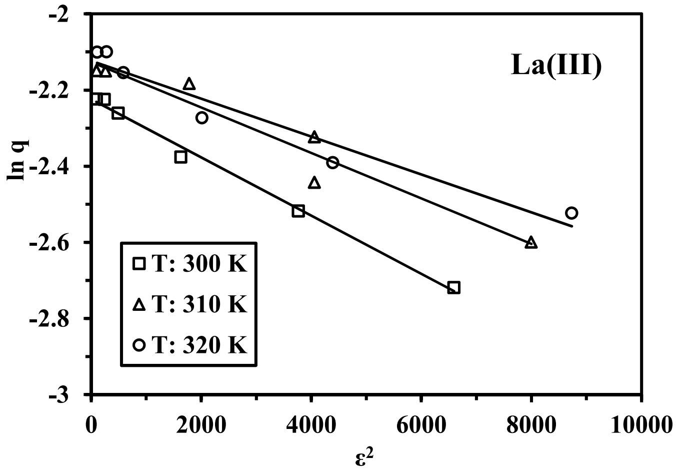

Figure S4. *Cont.*

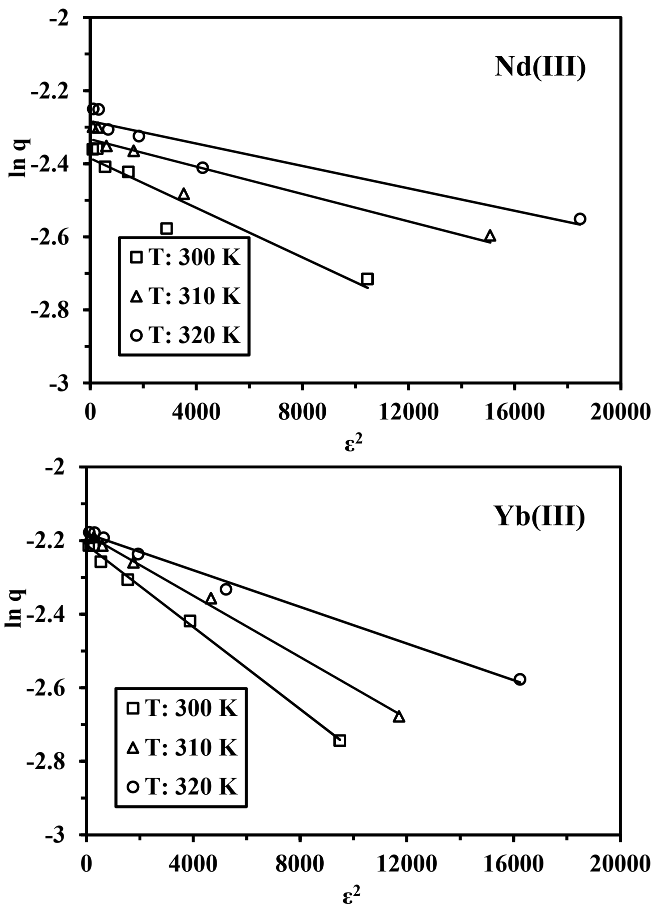

Figure S4. D-R isotherms plots for sorption of La(III), Nd(III) and Yb(III) ions at different temperatures.

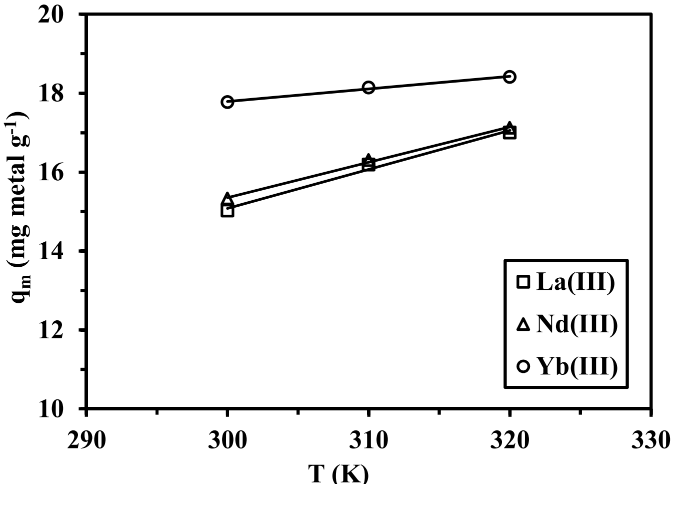

Figure S5. Plot of the values of q_e of La(III), Nd(III) and Yb(III) ions as a function of temperature. (t = 4 h, pH = 5, m = 0.05 g, V = 20 mL).
